# Unveiling the Unexpected: Co-occurrence of Acute Pancreatitis and Riedel’s Lobe

**DOI:** 10.7759/cureus.52325

**Published:** 2024-01-15

**Authors:** Andreea I Ghiță, Matei Olteanu, Alex E Debelka, Oana M Cîlțea, Mihai R Pahomeanu

**Affiliations:** 1 Faculty of Medicine, Carol Davila University of Medicine and Pharmacy, Bucharest, ROU; 2 Research, Bucharest Acute Pancreatitis Index (BUC-API) Study Group, Bucharest, ROU; 3 Faculty of Medicine, University of Oradea, Oradea, ROU; 4 Radiology, University Emergency Hospital of Bucharest, Bucharest, ROU; 5 Gastroenterology, University Emergency Hospital of Bucharest, Bucharest, ROU

**Keywords:** riedel's lobe, incidental, ct, acute pancreatitis, liver

## Abstract

Riedel's lobe is a rare anatomical variant of the liver, more often being diagnosed incidentally, with the patient being investigated for other underlying pathology. As regards acute pancreatitis, this represents one of the most treated diseases worldwide in gastroenterology with a variable severity and outcome. Here, we report a case of a non-palpable Riedel's lobe in a 47-year-old man, smoker, and chronic alcohol consumer, who presented to the hospital with epigastric pain radiating in the right hypochondrium, accompanied by nausea. Based on his clinical examination, laboratory, and imaging findings, he was admitted in the gastroenterology department with the diagnosis of alcohol-related acute pancreatitis. The computed tomography scan emphasized the presence of Riedel's lobe, causing an increased anterior diameter of the liver. Riedel's lobe is, in most cases, an unforeseen radiologic disclosure, which can remain clinically latent, or it can raise confusion regarding the differential diagnosis.

## Introduction

Riedel's lobe, named after a German surgeon in 1888, is a rare anatomical variation of the liver, often being described as a downward extension of its right lobe [1,2]. It is mostly discovered incidentally when a patient is being investigated for another underlying affliction, and it can remain either a benign discovery or lead to complications, even malignancy [3]. Since it can be easily overlooked, its prevalence is not clear yet, varying between 3.3% and 31% [4,5].

Acute pancreatitis is one of the most common inpatient diagnoses in gastroenterological ward in the world with a mortality estimated at 5.5% [6,7]. Most severe complications of acute pancreatitis include systemic inflammatory response syndrome (SIRS), pancreatic necrosis, penetration in the duodenum, or gastric wall. The incidence of this disease is rising, with an average of 3.07% per year [8], becoming a burden for the healthcare systems both medically and financially.

Here, we report a case of Riedel's lobe in a 47-year-old male diagnosed with alcohol-related acute pancreatitis.

## Case presentation

A 47-year-old without any medical history, male smoker (30 packs-year), and chronic alcohol consumer (average five to 10 units of alcohol daily) presented with epigastric pain radiating to the right hypochondrium and to the back, accompanied by nausea. The symptoms started approximately 12 hours before admission. The patient denied having any fever or chills.

On examination, cardiovascular examination showed a blood pressure of 150/10 mmHg, a heart rate of 110 beats per minute (rhythmic), normal heart sounds, and no cardiac murmurs. The patient was afebrile, with an ethylic tremor in the upper limbs, normal-colored integuments, and mucosa. Regarding the respiratory system examination, the patient had bilateral vesicular murmur without any rales or crepitus. Abdominal examination revealed a distended abdomen, with intense tenderness on palpation of the epigastrium and right hypochondrium. The liver had a prehepatic diameter of 180 mm, with a smooth and firm surface. The spleen and kidneys were not palpable, and diuresis was present.

Laboratory tests showed significant leucocytosis with neutrophilia, macrocytosis, increased levels of amylase and lipase, hepatic cytolysis, an inflammatory syndrome with increased fibrinogen, C-reactive protein and erythrocyte sedimentation rate, mild hepatic cholestasis, hypertriglyceridemia, decreased total iron-binding capacity, normal viral markers, and normal glycosylated hemoglobin (Table 1).

**Table 1:** Laboratory tests

Test	Pre-admission	1st day	4th day	Reference range
Amylase	207 U/L	91 U/L	74 U/L	15-115 U/L
Lipase	1293 U/L	434 U/L	271 U/L	73-393 U/L
Aspartate transaminase	100 U/L	51 U/L	53 U/L	2-40 U/L
Alanine transaminase	76 U/L	44 U/L	43 U/L	3-65 U/L
Total bilirubin	1.41 mg/dL	1.31 mg/dL	0.55 mg/dL	0-0.3 mg/dL
Direct bilirubin	0.47 mg/dL	0.57 mg/dL	0.22 mg/dL	0-1 mg/dL
Triglycerides	-	203 mg/dL	-	30-150 mg/dL
Fibrinogen	-	696 mg/dL	-	276-471 mg/dL
C-reactive protein	-	21.2 mg/dL	-	0-0.9 mg/dL
Erythrocyte sedimentation rate	-	60 mm/h	-	5-10 mm/h
Glucose	129 mg/dL	84 mg/dL	84 mg/dL	74-106 mg/dL
Glycosylated hemoglobin	-	5.9 g/dL	-	4.3-6 g/dL
White blood cells	-	18.4x10^3^/µL	9.95x10^3^/µL	3.98-10.8x10^3^/µL
Neutrophils (#)	-	15.3x10^3^/µL	6.79x10^3^/µL	2-6x10^3^/µL
Viral markers (HBsAg and anti-HCV Ab)	-	Negative	-	Negative
Total iron-binding capacity	-	247 mg/dL	-	250-250 mg/dL

Electrocardiogram findings included a sinus rhythm with a heart rate of 100 beats per minute, a QRS axis of 30°, minor right bundle branch block, and T-wave changes in leads D I, aVL, V1, and V4-V6.

Abdominal ultrasound revealed a normal-sized gallbladder without stones, non-dilated common and intrahepatic bile ducts, and a heterogeneous pancreas, hyperechoic without any visible collections and no visible fluid accumulation around the pancreas and in the posterior of the pouch of Douglas.

**Figure 1:**
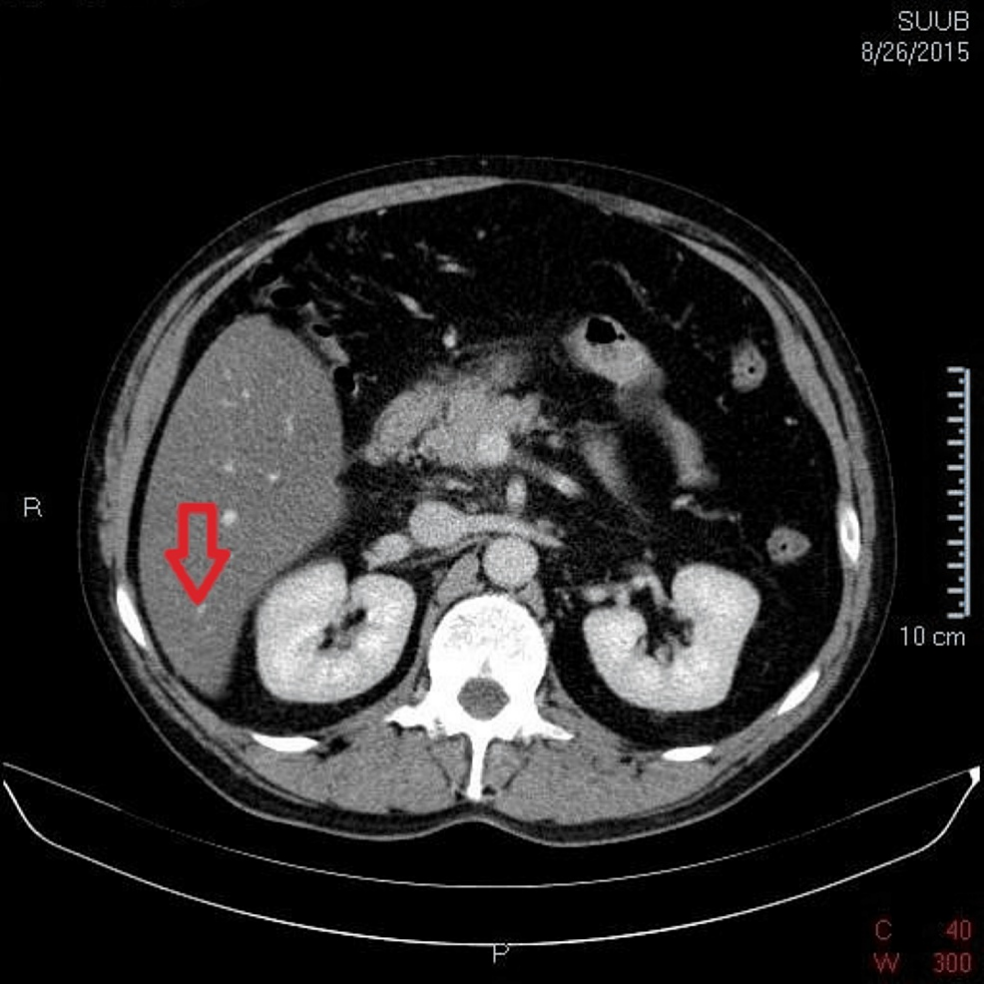
Riedel's lobe

**Figure 2:**
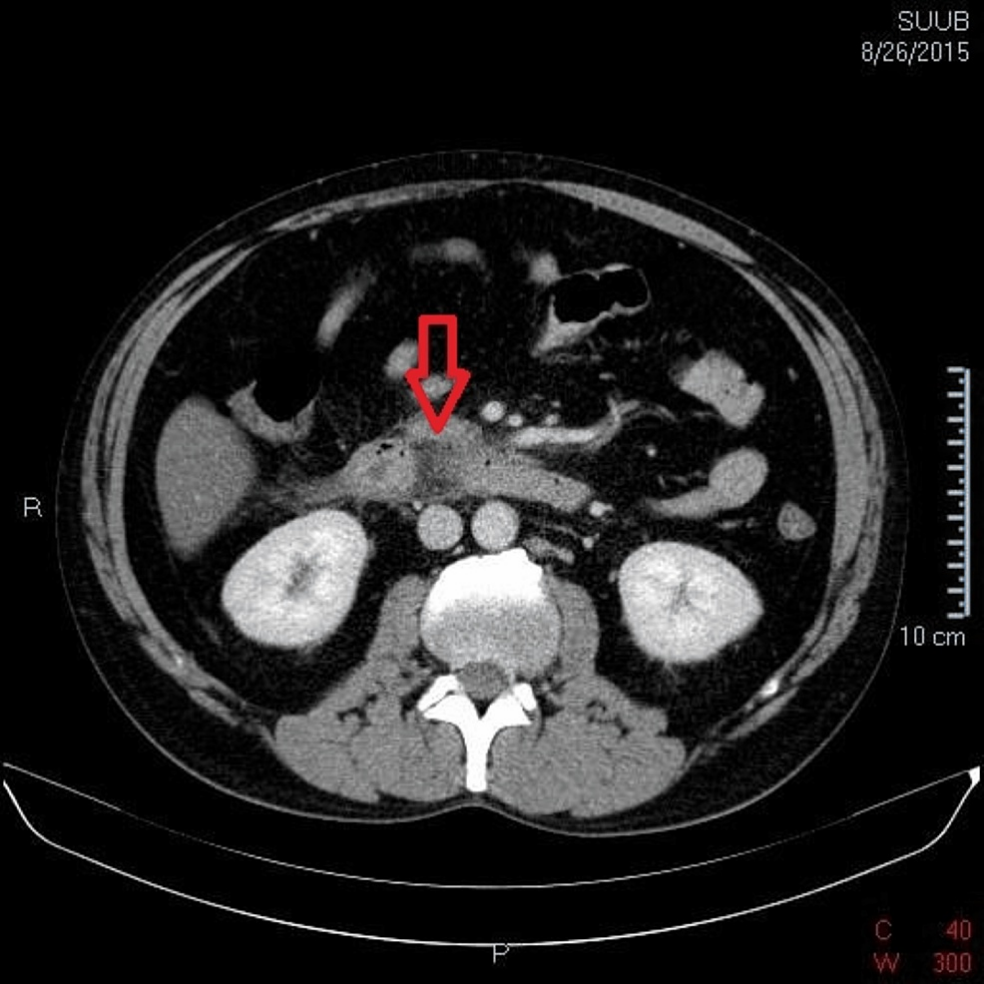
Typical CT-scan alterations in acute pancreatitis

The computed tomography scan of the abdomen, both native and post-contrast, revealed a liver with increased dimensions (cranio-caudal diameter 230 mm) and homogenous steatosis, along with a distinct Riedel's lobe (Figure 1). The pancreas had enlarged dimensions with a diffuse contrast in the cephalic region, with a notable thickening of the peripancreatic fat, associated with adjacent thin liquid streaks of about 5 mm thickness near the anterior right renal fascia (Figure 2). Moreover, the duodenum (second and third) presented thickened with oedematous walls. The gallbladder was distended with slender walls without any radiodense stones, and the common bile duct, intrahepatic bile ducts, and duct of Wirsung were non-dilated. In addition, the spleen, kidneys, and adrenal glands had a normal CT appearance, but there were some enlarged abdominal lymph nodes, with maximum dimensions up to 8 mm at the lumbar-aortic level. No pleural effusion was detected.

After reviewing the results, the patient was diagnosed with ethylic acute pancreatitis along with several secondary conditions: remitted hypovolemic shock, hypokalaemia, hypertriglyceridemia, alcoholic liver disease, and alcohol withdrawal syndrome, which developed 24-72 hours after admission with the patient having hand tremors, sweating, and tachycardia.

During the admission, the patient was given the following treatments: nil per os for 24 hours after admission and parenteral nutrition with glucose 10% 500 ml bid, as the patient did not tolerate the nasojejunal tube, and diet progression afterwards. For hydric repletion and correction of the potassium serum levels, we used Ringer’s solution 1.5 ml/kg/h with de-escalation in concordance with the heart rate, diuresis, and blood pressure. For pain control, we administered acetaminophen 500mg tid; nausea was handled with metoclopramide 10mg tid. To prevent stress gastritis, we used omeprazole 40 mg qd. To control the alcohol withdrawal syndrome, we employed diazepam 10 mg bid for the first 72 hours alongside with thiamine 200 mg tid and metoprolol succinate 50 mg to control the heart rate.

The patient's condition improved with intravenous fluid hydration, electrolyte balance, B-complex vitamins, proton pump inhibitors, prokinetics, painkillers, beta-blockers, and fasting. At discharge, the patient's abdominal pain and nausea had been resolved, the patient having a blood pressure of 120/80 mm Hg and a heart rate of 90 beats per minute.

## Discussion

Riedel's lobe is an uncommon anatomical variation that is most frequently discovered incidentally. The variation consists of a hypertrophy of hepatic segments V and VI [9,10]. The prevalence is variable, as stated before, and it depends on numerous factors, but the contrast between the percentages is probably being a result of an inconsistent anatomical definition and diagnostic procedure [11,12].

The exact aetiology of Riedel's lobe is not identified yet, but there are some hypotheses in the literature that are being presumed: it can be either a result of an unusual embryological evolution or an outcome of intraabdominal inflammation in some patients, even a result of traction by adherences formed due to abdominal surgery [9,11]. 

The clinical significance of Riedel's lobe is still in dispute, having a scarcity of cases reported in the literature regarding its involvement in abdominal pathologies. However, there has been a plethora of case reports revealing Riedel's lobe in patients when they were being thoroughly investigated for other afflictions. As stated in other papers, patients with accessory liver lobes, like Riedel's lobe, as described by some, can be more prone to develop pedunculated tumors of the liver or other complications, such as torsion or compression [12]. These kinds of mechanical events can affect the organs nearby the liver, but the incidence of such events remains unknown. As regards differential diagnosis, this downward hepatic extension can be easily confused with other liver diseases, such as cirrhosis or hepatic neoplasia, congestive heart failure, and right-sided pleural effusion [9]. Riedel's lobe, when discovered, does not require any treatment, unless there are complications present, so it has a good prognosis [9,13].

## Conclusions

In this case report, Riedel's lobe was a totally unexpected imagistic finding, which is consistent with most of the situations reported in the literature. It is not clear if Riedel's lobe played a part in the pathogenesis of the patient’s pancreatitis, but the synchronic presence was most likely coincidental. However, maybe, the obstruction of the biliary ducts or compression could be involved in pathology, although unlikely. Riedel's lobe requires further research to accurately establish the presence of clinical manifestations and the complications that can appear when it is present alongside other pathologies.
